# Interplay between Three RND Efflux Pumps in Doxycycline-Selected Strains of *Burkholderia thailandensis*

**DOI:** 10.1371/journal.pone.0084068

**Published:** 2013-12-27

**Authors:** Fabrice Vincent Biot, Mélanie Monique Lopez, Thomas Poyot, Fabienne Neulat-Ripoll, Sabrina Lignon, Arnaud Caclard, François Michel Thibault, Andre Peinnequin, Jean-Marie Pagès, Eric Valade

**Affiliations:** 1 Unité de Bactériologie/UMR_MD 1, Institut de Recherche Biomédicale des Armées, Brétigny-sur-Orge, France; 2 UMR_MD 1, Aix Marseille Université, IRBA, Facultés de Médecine et de Pharmacie, Marseille, France; 3 Pôle de génomique, Institut de Recherche Biomédicale des Armées, Brétigny-sur-Orge, France; 4 FR 3479 Plate-forme de Protéomique, CNRS, Aix-Marseille Université, Marseille, France; 5 Ecole du Val-de-Grâce, Paris, France; University of Technology Sydney, Australia

## Abstract

**Background:**

Efflux systems are involved in multidrug resistance in most Gram-negative non-fermentative bacteria. We have chosen *Burkholderia thailandensis* to dissect the development of multidrug resistance phenotypes under antibiotic pressure.

**Methodology/Principal Findings:**

We used doxycycline selection to obtain several resistant *B. thailandensis* variants. The minimal inhibitory concentrations of a large panel of structurally unrelated antibiotics were determined ± the efflux pump inhibitor phenylalanine-arginine ß-naphthylamide (PAßN). Membrane proteins were identified by proteomic method and the expressions of major efflux pumps in the doxycycline selected variants were compared to those of the parental strains by a quantitative RT-PCR analysis. Doxycycline selected variants showed a multidrug resistance in two major levels corresponding to the overproduction of two efflux pumps depending on its concentration: AmrAB-OprA and BpeEF-OprC. The study of two mutants, each lacking one of these pumps, indicated that a third pump, BpeAB-OprB, could substitute for the defective pump. Surprisingly, we observed antagonistic effects between PAßN and aminoglycosides or some ß-lactams. PAßN induced the overexpression of AmrAB-OprA and BpeAB-OprB pump genes, generating this unexpected effect.

**Conclusions/Significance:**

These results may account for the weak activity of PAßN in some Gram-negative species. We clearly demonstrated two antagonistic effects of this molecule on bacterial cells: the blocking of antibiotic efflux and an increase in efflux pump gene expression. Thus, doxycycline is a very efficient RND efflux pump inducer and PAßN may promote the production of some efflux pumps. These results should be taken into account when considering antibiotic treatments and in future studies on efflux pump inhibitors.

## Introduction

Bacteria can adapt to a wide range of environmental conditions. Antimicrobial compounds constitute environmental chemical stresses for bacterial cells and many pathogens have developed appropriate mechanisms conferring protection against this external attack. Active efflux plays a major role in this resistance, and multidrug efflux pumps decrease the accumulation of drugs within cells. The resistance-nodulation-division (RND) family of efflux pumps is ubiquitous in Gram-negative bacteria. The tripartite efflux pump complexes of this family are the major components of intrinsic multiresistance, which may complicate the treatment of infections due to these bacteria [1].

The genus *Burkholderia* is known for its numerous antimicrobial resistances and its multidrug-resistant phenotypes are often attributed to RND efflux pumps. Indeed, *Burkholderia* species have many RND efflux pumps in their genomes. The molecular basis of multiple drug resistance in the highly pathogenic *Burkholderia pseudomallei*, the etiological agent of melioidosis, is known [2]. The three principal RND efflux pumps involved in multidrug resistance in *B. pseudomallei* have been characterized: AmrAB-OprA, BpeAB-OprB, and BpeEF-OprC. Each RND efflux pump is encoded by an operon and is a tripartite complex: an integral inner transporter (AmrB, BpeB and BpeF respectively), a periplasmic adaptator named Membrane Fusion Protein (AmrA, BpeA and BpeE respectively) and the outer membrane channel named Outer Membrane Factor (OprA, OprB and OprC respectively) [3]–[9]. These complexes can export drugs outside the bacteria [10].

*B. thailandensis*, an environmental Gram-negative bacterium, is an opportunistic pathogen in immunocompromised patients [11], [12]. Due to its low level of virulence and its close relationship to *B. pseudomallei* and *Burkholderia mallei*, *B. thailandensis* is often used as a model organism in studies of the factors controlling both virulence and metabolism in the genus *Burkholderia* [13].

We have recently shown that *B. thailandensis* can become multidrug-resistant under chloramphenicol selection pressure, due to the overexpression of two RND efflux pumps [14]. These two pumps are very similar to the well characterized BpeAB-OprB and BpeEF-OprC pumps of *B. pseudomallei*. In this study, we used *B. thailandensis* as a model, to study the expression of RND efflux pumps induced by antibiotics in current use.

Doxycycline is one of the most widely used semisynthetic tetracyclines in clinical practice. It is well-tolerated and safe [15]. Due to its broad-spectrum antibiotic efficacy, doxycycline is indicated for the treatment of various infections, including anthrax, plague, brucellosis, tularemia, glanders and melioidosis [16], [17]. In Gram-negative bacteria, tetracycline, like chloramphenicol and imipenem, can induce multidrug resistance by triggering the overexpression of RND efflux pumps, such as the *acrAB* efflux system [18].

Specific mutants constructs have been used to determine the substrate selectivity of each efflux pump in *B. pseudomallei* and cyclines have been identified as the substrates of BpeAB-OprB and BpeEF-OprC [8], [19]. However, the connections between these pumps in wild-type strains remain unclear. Viktorov *et al.* showed that the spectrum of cross-resistance in *B. thailandensis* was similar to that in *B. pseudomallei*, by selecting strains on media containing a fluoroquinolone and a cephalosporin, and demonstrated that this multidrug resistance was associated with the overexpression of different RND efflux pumps [20].

Little is known about the role of doxycycline in the selection of multidrug-resistant strains of Gram-negative bacteria. We previously suggested that this antibiotic, like chloramphenicol, might induce the synthesis of efflux pumps [14]. The aim of this study was to assess the extent to which doxycycline was able to select a multidrug resistance phenotype of *B. thailandensis in vitro* and to dissect the mechanisms underlying this resistance. Through a combination of bacteriological, proteomic and transcriptomic analyses, we demonstrated that doxycycline was associated with an overexpression of various efflux pumps that are expressed at different levels, depending on the step of the antimicrobial selection, thereby revealing a reversible multidrug resistance phenotype.

## Materials and Methods

### Bacterial strains, growth media and selection of doxycycline-resistant strains

Bacteria were grown at 37°C in Luria–Bertani (LB) broth, in trypticase soy (TS) broth or on TS agar (Difco Laboratories, Detroit, MI, USA). *B. thailandensis* ATCC 700388, referred to here as E264 (type strain), was used as the wild-type strain. Four strains – E264DC16, E264DC32, E264DC64 and E264DC128 –were sequentially obtained from the reference strain, ATCC 700388, by culture on a gradient with concentration steps of 8–16, 16–32, 32–64, and 64–128 mg/L doxycycline (doxycycline hyclate, Sigma-Aldrich, MO, USA). The resulting strains, E264, E264DC16, E264DC32, E264DC64 and E264DC128, were routinely maintained on 0, 16, 32, 64 and 128 mg/L doxycycline, respectively, on TS agar. Two efflux pump-defective mutants of strain E264, lacking *amrRAB* and *bpe-oprC* (E264Δ*amrRAB* and E264Δ*bpeEF-oprC*), respectively, were obtained by collaboration with Thongdee *et al*. [21]. It is important to note that the mutant E264Δ*amrRAB* is deficient in *amrR* gene. This gene, encoding the repressor AmrR, is adjacent to the operon *amrAB-oprA*. The same method was applied to these two mutants, to generate four doxycycline-resistant variants for each mutant.

### Antibiotic susceptibility tests

The minimal inhibitory concentrations (MICs) of all the antibiotics tested were determined by the Etest® procedure (Biomérieux, France), on Mueller-Hinton II agar. Results were read after incubation for 24 h at 37°C and are expressed in mg/L. The efflux pump inhibitor, MC 207,110 or phenylalanine-arginine ß-naphthylamide (PAßN) (Sigma-Aldrich Chimie, Saint-Quentin Fallavier, France), was used as previously described [14], [22]. PAßN (50 and 200 mg/L) was incorporated into Mueller Hinton II agar to obtain the final concentration indicated.

### Preparation of membrane fractions

Bacterial membrane fractions were prepared from 50 mL mid-exponential phase cultures in LB broth. Bacteria were harvested, washed and resuspended in 10 mL of cold sodium phosphate buffer (100 mM NaH_2_PO_4_/Na_2_HPO_4_, pH 7.4) containing 1 mg/mL lysozyme. Cells were lysed in a FastPrep FP120 high-speed benchtop homogenizer, in Lysing Matrix E tubes (MP Biomedicals France; 10 cycles; 6 m/s; 40 s per cycle) and intact bacteria were removed by centrifugation (10,000 *g*; 10 min; 4°C). Whole membranes were recovered from the supernatant by ultracentrifugation (40,000 *g*; 60 min; 4°C) and were incubated in 0.15% sodium N-laurylsarcosinate for 30 min at room temperature, to extract the detergent-soluble material, in a modified version of a previously described procedure [14], [23]. The insoluble membrane fractions were pelleted by centrifugation (40,000 *g*; 60 min; 20°C). Pellets were resuspended in solubilization buffer (NuPAGE® LDS Sample Buffer + NuPAGE® LDS Sample Reducing Agent, Invitrogen™, Villebon-sur-Yvette, France) and heated for 5 min at 95°C, as previously described [24].

### SDS-PAGE

The samples of membrane fractions prepared above were run on SDS-polyacrylamide gels (NuPAGE® Novex® Bis-Tris Mini Gels 10%, 1.0 mm*10 well, MOPS SDS Running Buffer, Invitrogen™, Villebon-sur-Yvette, France). After migration, gels were stained with Coomassie Brillant Blue G (Euromedex, France).

### Protein identification: peptide digestion and nano-electrospray MS/MS identification

Protein bands were excised from gels and digested in the resulting gel plugs with sequencing-grade modified porcine trypsin (Promega, Madison, WI). The peptides were extracted (0.1% trifluoroacetic acid), dried under vacuum and redissolved in loading buffer (98% H2O, 2% acetonitrile, 0.05% trifluoroacetic acid).

For nano-electrospray MS/MS identification, we used a 2-D LC coupled to a dynamic nano spray ionization source on the ion-trap an LCQ™ Deca XP Plus ion trap mass spectrometer (Thermo Finnigan). A peak list was generated by Bioworks Browser version 3.3 (Thermo Electron) using the following parameters: MW range 300–3500, threshold absolute, precursor ion tolerance 1.40 amu, group scan 2, minimum ion count 1, minimum group count 15, considering singly, doubly and triply charged ions. Protein identification was performed by the Sequest (v28 rev12) algorithm using the non-redundant NCBI database (http://www.ncbi.nlm.nih.gov) restricted to *B. thailandensis* (17457 entries). Criteria for positive identification of peptides were assessed by a cross-correlation number (Xcorr) versus charge state [25].

Protein identification was taken into account when presenting at least two unique peptides (Peptide Hits) of rank 1 (Protein score>20) and probability (P) below 10^−3^. The positive matches with two unique peptides or with probability above 10^−3^ were manually checked and only MS/MS spectra [25].

### RNA isolation and reverse transcription

Strains were cultured in 5 mL of LB broth with the appropriate concentration of doxycycline (0, 16, 32, 64, 128 mg/L) or in presence of PAßN (50 or 200 mg/L) until mid-exponential phase (OD*_600_* = 0.6). All extractions were performed four times for each strain and each growth condition.

A 1 mL aliquot of each culture, corresponding to 5×10^7^ cells, was added to 2 mL of RNAprotect Bacteria Reagent (Qiagen, Courtaboeuf, France). Total RNA was isolated with the RNeasy lipid tissue minikit, according to the manufacturer's instructions, by a silica-based method. Genomic DNA was eliminated by incubation with RNase-free DNase I (Qiagen, Courtaboeuf, France) treatment during the isolation procedure. Finally, RNA was eluted in a volume of 60 µL of RNase-free water. Total RNA concentration and purity were determined at neutral pH by spectrophotometric analysis (Nanodrop 1000, ThermoFisher Scientific).

Reverse transcription was performed with a Reverse Transcription Core Kit (Eurogentec France S.A.S.U., Angers), according to the manufacturer's instructions, with 600 ng of RNA, 2.5 µM random nonamers and RNase inhibitor (12 U), in a final volume of 30 µL. cDNA was synthesized with a constant volume of total RNA extract, to minimize RT-qPCR variability due to differences between samples [26]. For this purpose, RNA extract (≤600 ng total RNA) was supplemented with yeast tRNA (Ambion) to obtain 600 ng of RNA in total. The volume of RNA extract processed was identical to that of the most concentrated sample, which contained 600 ng of total RNA. The sample was initially heated at 25°C for 10 min, then reverse transcription was carried out at 48°C for 30 min, and the reverse transcriptase was inactivated by heating at 95°C for 5 min. The cDNA was finally stored at −80°C.

### Real-time PCR

For standard DNA assays, primers were designed and optimized and their specificity was confirmed as previously described [27]. The sequences of the genes studied were obtained from GenBank and the primers were designed and optimized with MacVector® software (version 11.1.2; Accelerys, SanDiego, USA). The sequences of the primers are listed in Table S1. Primer pairs were tested on a 4-log calibration curve from recombinant product. Maximal and minimal Cq obtained (Cq max and min) and the obtained efficiencies are shown in Table S1. Quantitative PCR (qPCR) were carried out with the LightCycler Fast Start DNA Master Sybr Green Kit (Roche Applied Science, Mannheim, Germany), with 0.25–0.5 µl of cDNA in a final volume of 20 µl containing 4 mM MgCl_2_ and 0.4 µM of each primer (final concentration). qPCR was performed in a LightCycler® apparatus (Roche Applied Science, Mannheim, Germany), with 50 cycles of 95°C for 20 s (denaturation) and 53–58°C for 2–8 s [annealing temperature and time, which are primer and structure-dependent (Table S1)] followed by a final step of 3–5 s at 72°C (elongation). No template controls were used to test the specificity of the primers pair and a melting curve analysis was performed. The quantification cycles (Cq) were calculated using Light Cycler Software v.3.5 (Roche Applied Science). Quantification was assessed using the exponentiated values of Cq [28]. All the Cq values were below the lower limit of quantification, unless the gene was defective (data not shown). Quantification was achieved relative to the geometric mean of four internal validated control genes – *rumA* 23S rRNA, *rimM* 16S rRNA, *dnaK* and *rpsL 30S* – after gene stability had been assessed with the geNorm algorithm [29]. Mean values (± standard error of the mean) for mRNA levels obtained in four independent biological replicate experiments and analyzed by qPCR were considered. The four biological replicates were standardized as described by Willems *et al*. [30].

### Statistical analysis

At least four biological replicates were used for statistical analyses, based on nonparametric Mann-Whitney *U* tests. Levels of gene transcription are expressed as ratios with respect to the values for the wild-type strain E264 (set at 1). All values shown are means ± SEM. We considered *p*-values below 0.05 to be significant.

## Results

### Antibiotic susceptibility of *B. thailandensis* variants

*B. thailandensis* strain ATCC 700388 was grown in the presence of doxycycline (0.5–128 mg/L) and successive doxycycline-resistant derivatives were obtained. We retained only four of these derivatives: E264DC16, E264DC32, E264DC64 and E264DC128, selected by growing the wild-type strain ATCC 700388 (E264) on medium containing 16, 32, 64 and 128 mg/L doxycycline, respectively. The MICs of various antibiotics were determined for these strains and compared with those for the parental susceptible strains (Tables 1 and 2).

**Table 1:** Susceptibility of *B. thailandensis* strains to cyclines, and antibiotics from other classes.

B. thailandensis strains	MIC (mg/L)										
	Cyclines				Quinolones						
	DOX	TET	MIN	TGC	NAL	CIP	LVX	CHL	SXT	ERY	CST
**E264 (WT)**	1.5	2	1.5	1	12	1	2	4	3	>256	>256
**E264DC16**	8	4	6	3	16	2	3	3	3	>256	>256
**E262DC32**	16	6	6	6	32	3	3	8	2	>256	>256
**E264DC64**	>256	32	64	32	>256	>32	>32	>256	>32	>256	>256
**E264DC128**	>256	32	64	32	>256	>32	>32	>256	>32	>256	>256

Antimicrobial agent abbreviations: DOX, doxycycline; TET, tetracycline; MIN, minocycline; TGC, tigecycline; CHL, chloramphenicol; NAL, nalidixic acid; CIP, ciprofloxacin; LVX, levofloxacin; SXT, trimethoprim/sulfamethoxazole; ERY, erythromycin; CST, colistin.

**Table 2:** Susceptibility of *B. thailandensis* strains to ß-lactams and aminoglycosides.

B. thailandensis strains	MIC (mg/L)																				
	ß-lactams																Aminoglycosides				
	AMX	AMC	OXA	TIM	TMO	PIP	TZP	CEF	CXM	CAZ	CTX	ATM	IPM	ETP	MEM	DPM	AMK	GEN	TOB	KAN	NET
**E264 (WT)**	16	4	>256	1.5	6	1.5	2	>256	16	1	6	48	0.25	2	0.5	0.5	128	64	16	16	48
**E264DC16**	16	6	>256	2	8	1.5	2	>256	16	1	6	48	0.19	2	0.5	0.5	>256	96	128	256	64
**E262DC32**	16	6	>256	1.5	6	1	1.5	>256	12	1	8	32	0.25	2	0.38	0.38	>256	128	256	256	64
**E264DC64**	16	1.5	>256	0.75	3	0.38	1	>256	4	0.5	1.5	16	0.125	1.5	0.19	0.125	64	48	12	12	24
**E264DC128**	8	2	>256	0.75	3	0.38	1.5	>256	4	0.5	1.5	16	0.125	1	0.125	0.125	64	48	12	12	16

Antimicrobial agent abbreviations: AMX, amoxicillin; AMC, amoxicillin/clavulanate (2/1); OXA, oxacillin; TIM, ticarcillin/clavulanic acid (2 mg/L); TMO, temocillin; PIP, piperacillin; TZP, piperacillin/tazobactam (4 mg/L); CEF, cefalotin; CXM, cefuroxime; CAZ, ceftazidime; CTX, cefotaxime; ATM, aztreonam; IPM, imipenem; ETP, ertapenem; MEM, meropenem; DPM, doripenem; AMK, amikacin; GEN, gentamicin; TOB, tobramycin; KAN, kanamycin; NET, netilmicin.

Many of these MICs showed changes in the derivative variants, particularly in E264DC64 and E264DC128, selected at the highest concentrations. All resistant variants had a reduced susceptibility to doxycycline and to other cyclines (tetracycline, minocycline, tigecycline), but also to structurally unrelated antibiotics as quinolones (nalidixic acid, ciprofloxacin, levofloxacin) (Table 1). Susceptibility to chloramphenicol and trimethoprim/sulfamethoxazole was only reduced in E264DC64 and E264DC128. It seems that the level of resistance in the four variants was not directly associated with the step-by-step increase in doxycycline concentration. E264DC16 and E264DC32 showed only a 2–3 fold increase in their MICs of cyclines and quinolones compared to the parental strain, while MICs of these antibiotics increased between 16–170 fold for E264DC64 and E264DC128.

Surprisingly, a small but systematic decrease in MICs, especially in E264DC64 and E264DC128, was obtained for numerous ß-lactams (amoxicillin/clavulanate, piperacillin, cefuroxime, ceftazidime, cefotaxime, aztreonam, imipenem, ertapenem, meropenem, doripenem) (Table 2).

A paradoxical response was obtained for aminoglycosides, with an increase in MICs for the first two levels (E264DC16, E264DC32) and then decrease for the last two levels (E264DC64, E264DC128) of selection, for the five aminoglycosides tested.

### Effect of PAßN on antibiotic susceptibility

RND efflux pumps are involved in many multidrug resistance phenotypes. They are therefore potential targets for new antibacterial agents that could restore susceptibility to many drug classes [31]. PAßN has been implicated in the blocking of RND-family efflux system activity and the reversal of multidrug resistance in Enterobacteriaceae, such as *Klebsiella pneumoniae* and *Enterobacter aerogenes*, and in non fermentative Gram-negative bacteria, such as *Pseudomonas aeruginosa*, *Acinetobacter baumannii* and *Burkholderia cepacia* [22], [24], [32], [33], [34], [35]. We have previously shown that PAßN is most effective as an efflux pump blocker for *B. thailandensis* when used at a concentration of 200 mg/L [14]. We therefore used two concentrations (50 and 200 mg/L) in this study.

PAßN partly restored the activities of doxycycline and nalidixic acid especially in strains E264DC64 and E264DC128, that showed an 8–16 fold decrease in the MICs of this both antibiotics, but these MICs were 2–16 fold higher than the MICs observed in the parental strain. However, the activity of PAßN seemed to be the most important with trimethoprim/sulfamethoxazole whose MICs decreased more than 32 fold in E264DC64 and E264DC128 (Table 3). In presence of 200 mg/L PAßN, MICs of trimethoprim/sulfamethoxazole in all strains were lower than the MIC of this antibiotic used alone in the parental strain (3–12 fold). By contrast to our findings for doxycycline, nalidixic acid and trimethoprim/sulfamethoxazole, no restoration of susceptibility was observed for chloramphenicol.

**Table 3:** Effects of PAßN on the antibiotic susceptibility of *B. thailandensis* strains.

	MIC (mg/L)															
**Antibiotic**	**DOX**			**SXT**			**NAL**		**CHL**		**TGC**		**CAZ**		**CTX**	
**PAßN (mg/L)**	**0**	**50**	**200**	**0**	**50**	**200**	**0**	**200**	**0**	**200**	**0**	**200**	**0**	**200**	**0**	**200**
**E264 (WT)**	1.5	3	2	3	0.38	0.25	12	4	4	3	1	3	1	1	6	8
**E264DC16**	8	24	12	3	0.5	0.25	16	4	3	3	3	12	1	1	6	8
**E264DC32**	16	24	12	2	0.38	0.25	32	8	8	8	6	12	1	1	8	12
**E264DC64**	>256	32	24	>32	3	1	>256	24	>256	>256	32	16	0.5	0.8	2	8
**E264DC128**	>256	32	16	>32	2	1	>256	32	>256	>256	32	16	0.5	0.8	2	8
**Antibiotic**	**CIP**			**GEN**			**NOR**		**AMK**		**AMC**		**IPM**		**ATM**	
**PAßN (mg/L)**	**0**	**50**	**200**	**0**	**50**	**200**	**0**	**200**	**0**	**200**	**0**	**200**	**0**	**200**	**0**	**200**
**E264 (WT)**	1	1.5	1	64	>1,024	>1,024	6	6	128	>256	4	8	0.25	0.5	48	48
**E264DC16**	2	2	2	96	>1,024	>1,024	12	12	>256	>256	6	6	0.19	0.4	48	64
**E264DC32**	3	3	3	128	>1,024	>1,024	24	16	>256	>256	6	6	0.25	0.4	32	64
**E264DC64**	>32	>32	>32	48	512	384	>256	48	64	>256	2	4	0.13	0.3	16	32
**E264DC128**	>32	>32	>32	48	384	256	>256	64	64	>256	2	6	0.13	0.3	16	32

Abbreviations: PAßN, phenylalanine-arginine ß-naphthylamide; DOX, doxycycline; SXT, trimethoprim/sulfamethoxazole; NAL, nalidixic acid; CHL, chloramphenicol; TGC, tigecycline; CAZ, ceftazidime; CTX, cefotaxime; CIP, ciprofloxacin; GEN, gentamicin; NOR, norfloxacin; AMK, amikacin; AMC, amoxicillin/clavulanate (2/1); IPM, imipenem; ATM, aztreonam.

Surprisingly, an antagonistic effect was observed when PAßN was used in combination with aminoglycosides (gentamicin and amikacin) or ß-lactams (cefotaxime, amoxicillin/clavulanate, imipenem and aztreonam), with an increase in MICs observed in the wild-type strain and doxycycline-resistant derivatives: 2–4 fold for ß-lactams and 2–16 fold for aminoglycosides.

### SDS-PAGE analysis of membrane fractions from the various *B. thailandensis* strains

**Figure 1:**
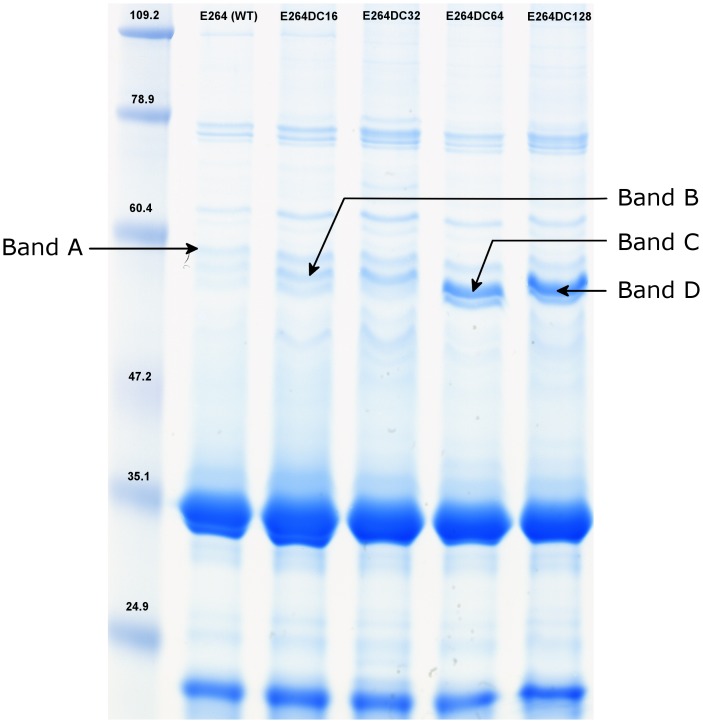
Analyses of the detergent-insoluble membrane proteins of doxycycline-resistant variants. SDS-PAGE analysis was performed on the E264 wild-type strain of *B. thailandensis*, and on the four doxycycline-resistant derivative strains, E264DC16, E264DC32, E264DC64 and E264DC128. Proteins were stained with Coomassie blue. The variants presented additional bands at around 58 kDa (band A), 55 kDa (band B) and at 54 kDa (bands C and band D). Molecular weight standards are indicated in kilodaltons.

The proteins present in the detergent-insoluble membrane fractions of the four resistant strains and the parental strain were analyzed by SDS-polyacrylamide gel electrophoresis (Figure 1). Staining of the protein bands for the resistant derivatives revealed significant variation in the 53 to 56 kDa region. In this region, the proteins corresponding to bands B, C and D seemed to be more abundant in the membrane fractions of the resistant variants than in those of the parental strains (Figure 1). Protein A (∼58 kDa) appeared to be present in similar amounts in the parental strain and the resistant variants. For the first resistant variants selected, E264DC16 and E264DC32, band B was more intense than that of the parental strain E264. Bands C and D were detected only in the resistant strains selected at the two highest concentrations of doxycycline, E264DC64 and E264DC128. These differences in band intensities were reproducibly observed in several independent SDS-PAGE analyses. The corresponding proteins were excised from the gels and identified by mass spectrometry.

### Identification of outer membrane proteins overproduced in the resistant variants

The four bands, A to D, were excised from the gels, and the proteins were retrieved and used for mass spectrometry determinations. The four proteins were identified as outer membrane channels of RND efflux pumps. These pump components have not previously been described in *B. thailandensis*, but their homologs in *B. pseudomallei* have been characterized. We recently showed that two of these pumps recognize the same substrates as their counterparts in *B. pseudomallei* [14].

The mass spectrum of band A (58 kDa) matched that of BTH_I0682 (58,312 Da) in the NCBR (GenBank) database, an outer membrane channel in *B. thailandensis* strain E264 (Table 4) [36]. A similar protein (97% identical) from strain K96243 of *B. pseudomallei* has been named OprB and corresponds to the outer membrane channel of the RND efflux pump BpeAB-OprB. To avoid confusion and, given the very close similarity between the two pumps, this name is currently used to designate BTH_I0680-BTH_I0681-BTH_I0682 in *B. thailandensis* strain E264 [4]. In *P. aeruginosa* PAO1, the homolog of BTH_I0682 is OprM (56% amino-acid sequence identity) [37].

**Table 4:** Identification of three outer membrane channels of RND efflux pumps.

Band no.	Accession no.	Protein	Mol. wt (Da)	Protein score	P	% coverage	Peptide (Hits)	Homologs in *B. pseudomallei* K96243 (% identity)	Homologs in *P. aeruginosa* PAO1 (% identity)
A	GI?257142607	BTH_I0682	58,312	30.19	5.27E-05	11.00	4	OprB (97%)	OprM (56%) OprJ (45%)
B	GI|257139187	BTH_I2443	55,378	150.18	3.78E-08	31.50	27	OprA (91%)	OprM (45%) OprJ (45%)
C	GI|257140923	BTH_II2104	54,999	240.23	1.19E-08	56.90	68	OprC (97%)	OprM (34%)
	GI|257139187	BTH_I2443	55,378	90.17	1.10E-08	21.70	11	OprA (91%)	OprM (45%) OprJ (45%)
	GI|257142607	BTH_I0682	58,312	50.19	2.36E-05	14.40	5	OprB (97%)	OprM (56%) OprJ (45%)
D	GI|257140923	BTH_II2104	54,999	190.27	2.52E-09	42.70	55	OprC (97%)	OprM (34%)
	GI|257139187	BTH_I2443	55,378	80.16	4.21E-05	14.20	10	OprA (91%)	OprM (45%) OprJ (45%)

Band B (55 kDa) was identified as BTH_I2443 (55,378 Da), another outer membrane channel of an RND efflux pump. This component is the homolog of OprA of *B. pseudomallei* strain K96243, a subunit of the AmrAB-OprA efflux pump (Table 4) [3]. The name AmrAB-OprA (amr: aminoglycoside and macrolide resistance) has already been used for *B. thailandensis* (BTH_I2445-BTH_I2444-BTH_I2443) [21]. OprA (BTH_I2443) was more abundant in the membrane fraction of the resistant strains E264DC16 and E264DC32 than in the parental strains and the other two resistant strains, E264DC64 and E264DC128.

Bands C and D (54 kDa) were identified as corresponding to BTH_II2104 (54,999 Da). BTH_II2104 is the homolog of OprC in *B. pseudomallei*, described as the outer membrane channel of the RND efflux pump BpeEF-OprC (BTH_II2106-BTH_II2105-BTH_II2104 in *B. thailandensis* strain E264 [6], [21]).

OprC was not detected in either the parental strain E264 or in the resistant variants E264DC16 and E264DC32, but it was found in E246DC64 and E264DC128.

### Antibiotic susceptibility of the RND-deficient mutants and their derivative doxycycline-resistant strains

E264Δ*amrRAB* and E264Δ*bpeEF-oprC* were constructed by Thongdee *et al.* [21]. These two strains were selected on doxycycline, with the same protocol applied to the parental strain E264, and the extent to which PAßN could reverse drug resistance was assessed. These two RND-deficient mutants displayed multidrug resistance at different successive steps during the doxycycline selection procedure (Table 5). E264Δ*amrRAB* was susceptible to gentamicin and erythromycin, and its doxycycline-resistant derivative strains remained susceptible to gentamicin but gradually became resistant to erythromycin (Table 5). In the presence of PAßN, a partial restoration of susceptibility was observed for doxycycline, chloramphenicol and nalidixic acid, but not for trimethoprim/sulfamethoxazole. No antagonistic effect was observed with PAßN. Doxycycline-resistant derivative strains of E264Δ*bpeEF-oprC* displayed multidrug resistance, with an increase in MICs not only for doxycycline, chloramphenicol, quinolones and fluoroquinolones, but also for aminoglycosides, by contrast to the results for strains selected from the parental wild-type strain E264 under doxycycline selection pressure. PAßN partially decreased the resistance of these strains to doxycycline, chloramphenicol and nalidixic acid (Table 5).

**Table 5:** Effects of PAßN on the antibiotic susceptibility of *B. thailandensis* strains and mutants.

B. thailandensis strains	DOX		CHL		NAL		SXT		AMC		GEN		ERY	
	PAßN 200 mg/L		PAßN 200 mg/L		PAßN 200 mg/L		PAßN 200 mg/L		PAßN 200 mg/L		PAßN 200 mg/L		PAßN 200 mg/L	
	−	+	−	+	−	+	−	+	−	+	−	+	−	+
E264 (WT)	1.5	2	4	3	12	4	3	0.25	4	8	64	>1,024	>256	>256
E264ΔamrRAB	0.19	0.38	2	2	4	3	2	2	4	12	1	1.5	4	12
E264ΔamrRABDC16	32	24	64	24	48	12	3	3	12	8	0.75	0.75	64	96
E264ΔamrRABDC32	32	24	48	32	32	12	4	4	8	12	0.75	0.75	96	96
E264ΔamrRABDC64	>256	64	64	64	32	16	>32	>32	12	16	0.75	0.75	192	>256
E264ΔamrRABDC128	>256	48	64	48	64	24	>32	>32	4	6	0.5	0.5	>256	>256
E264ΔbpeEF-oprC	1.5	1.5	4	4	8	4	2	2	3	6	128	384	>256	>256
E264ΔbpeEF-oprCDC16	16	8	16	8	16	2	2	0.75	12	32	256	256	>256	>256
E264ΔbpeEF-oprCDC32	48	32	12	6	16	16	>32	>32	6	8	512	384	>256	>256
E264ΔbpeEF-oprCDC64	>256	128	32	24	32	16	>32	>32	6	8	>1,024	768	>256	>256
E264ΔbpeEF-oprCDC128	>256	64	32	16	48	16	32	>32	12	8	384	512	>256	>256

Abbreviations: PAßN, phenylalanine-arginine ß-naphthylamide; DOX, doxycycline; CHL, chloramphenicol; NAL, nalidixic acid; SXT, triméthoprime/sulfamethoxazole; AMC, amoxicillin/clavulanate (2/1); GEN, gentamicin; ERY, erythromycin.

### Reversal of antimicrobial resistance

We investigated the stability of the resistance, by plating the parental wild-type strain E264 and its four doxycycline-resistant derivative strains on antibiotic-free medium. After eight subcultures, we determined the MICs of various structurally unrelated antibiotics (Table 6). The MICs in the strains initially resistant were similar to those for the parental strain. For doxycycline, MICs were only slightly higher than in the wild-type strain. These results suggest that doxycycline selected *B. thailandensis* strains expressing a mechanism of multidrug resistance that is not maintained in the absence of drug stress.

**Table 6:** Antibiotic susceptibility of the *B. thailandensis* strains after plating eight times on medium without doxycycline.

B. thailandensis strains	MIC (mg/L)					
	DOX	CHL	SXT	NAL	AMK	AMX
**E264 (WT)**	1.5	3	2	12	>256	16
**E264DC16**	6	3	2	12	>256	16
**E264DC32**	4	3	2	12	>256	24
**E264DC64**	6	4	2	24	>256	16
**E264DC128**	4	4	1.5	16	>256	24

Abbreviations: DOX, doxycycline; CHL, chloramphenicol; SXT, triméthoprime/sulfamethoxazole; NAL, nalidixic acid; AMK, amikacin; AMX, amoxicillin.

### RND efflux pump expression

**Figure 2:**
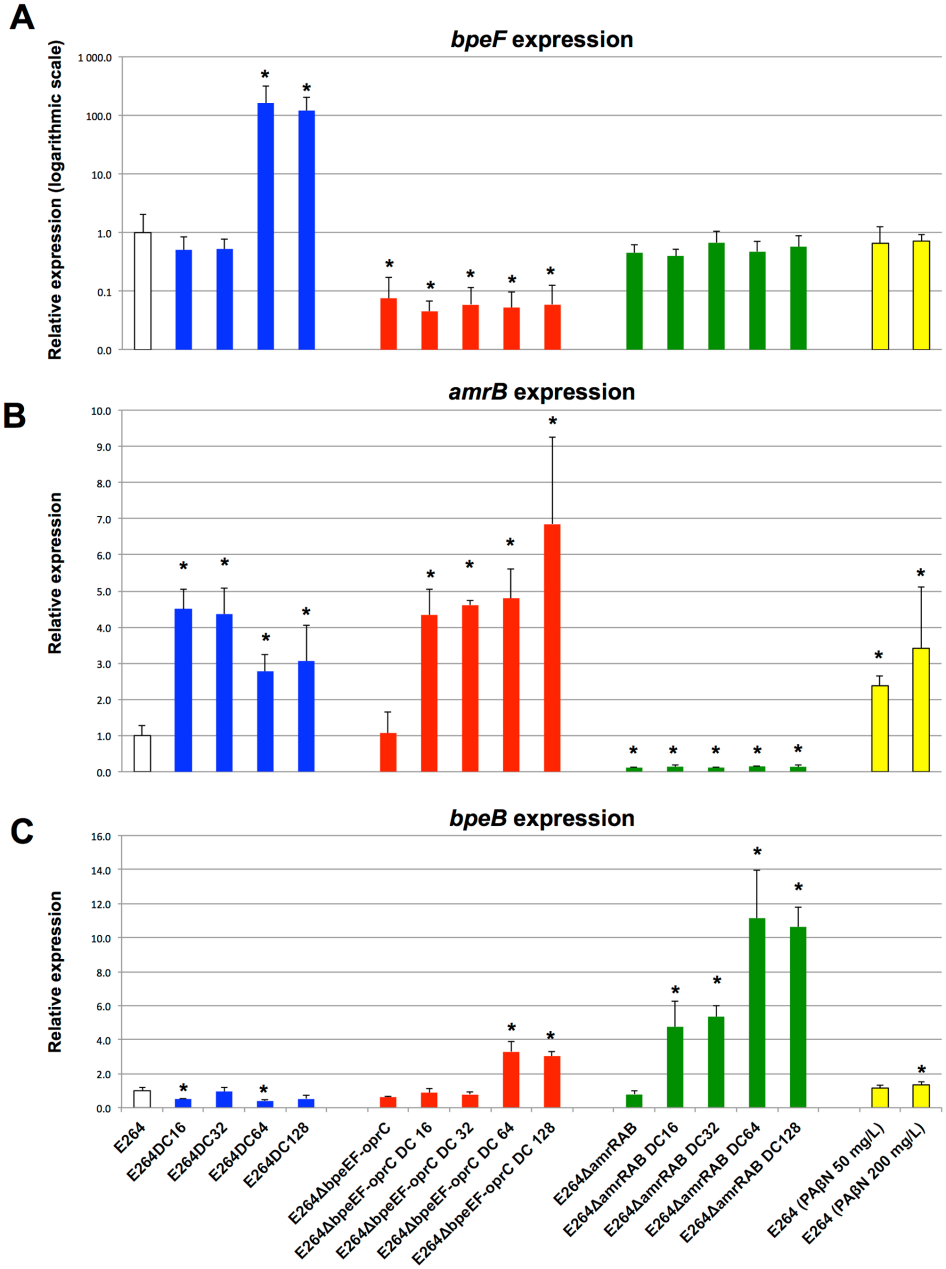
Quantitative RT-PCR analysis of *bpeF* (Figure 2A), *amrB* (Figure 2B) and *bpeB* (Figure 2C) gene expression. Expression levels were normalized with respect to the four reference genes previously selected, with wild-type strain E264 levels set to 1. Values are presented as means ± SEM of four biological replicates and are compared with the value for the wild-type strain E264. Significant differences are indicated by a single asterisk indicating a *p*-value<0.05 (Mann-Whitney *U* tests). Error bars indicate one standard error of the mean.

For all strains studied, we observed a modulation of the expression of the tested efflux components. In particular, the two strains selected on the highest concentrations of doxycycline displayed high levels of *bpeF* transcription (Figure 2A). We also observed the overexpression of *amrB* and *bpeF*, whose expression level was modulated according to the concentration of antibiotic used for selection, with *amrB* first increasing by a factor of 4.5 with respect to the wild-type strain E264 (Figure 2B) and *bpeF* expression then increasing by a factor of 140 (Figure 2A). There was also a significant difference in *amrB* transcription between the E264DC16/E264DC32 and E264DC64/E264DC128 groups (*p*<0.001) (Figure 2B), indicating that the expression of *amrB* decreased after the induction of *bpeF* gene transcription. We observed an increase in *bpeB* gene transcription in E264Δ*amrRAB* derivative strains (Figure 2C; *p*<0.05). In E264Δ*bpeEF-oprC* doxycycline-resistant derivative strains, an increase in *amrB* expression was observed, followed by an increase in *bpeB* (*p*<0.05).

PAßN stimulated the transcription of *amrB* and *bpeB* (*p*<0.05), in a dose-dependent manner, what was not observed for *bpeF*. The level of expression of *amrB* with 200 mg/L PAßN was higher than those obtained in the presence of 64 and 128 mg/L doxycycline. However, the expression of *bpeB* was only slightly increased in comparison with *amrB*, and that was only higher when 200 mg/L PAßN was used.

We also observed that expression level of *amrB* and *bpeB* was completely reversed in the variant E264DC128 when doxycycline is removed from the growth medium that was less the case for the pump *bpeF* (data not shown). The mutant E264Δ*amrRAB* and its derivative resistant variants, showed that the induction by doxycycline of the expression of the operon *amrAB-oprA* was independent of the presence of the repressor gene *amrR* (data not shown). It had already been shown in a strain of *B. pseudomallei* lacking *bpeR*, that *bpeAB-oprB* expression remained inducible by erythromycin to levels comparable to that of the wild-type parental strain [5]. Trunck *et al.* demonstrated that *amrAB-oprA* expression in gentamicin selected strains of *B. pseudomallei* was not due to promoter-up mutations or other *amrR* mutation [7]. Our results confirm that doxycycline is also capable of inducing the expression of *amrB* regardless of the presence of a mutation in the repressor gene *amrR*.

## Discussion

Doxycycline is currently used as an oral antibiotic for the treatment of infections with a broad spectrum of intracellular bacteria. We show here that this molecule can induce RND efflux pump expression in a Gram-negative bacterium and select multidrug-resistant strains. Another antibiotic of the cycline class, tetracycline, has been demonstrated to induce RND efflux pump overexpression in *Yersinia pestis* [18] and in *P. aeruginosa* [38]. Furthermore, some antibiotics, including chloramphenicol and imipenem, are known to select strains of Gram-negative bacteria displaying multidrug-resistance due to the overexpression of RND efflux pumps [14], [23], [24].

We provide here the first demonstration of the selection, by doxycycline, of *B. thailandensis* strains overproducing active efflux pumps. This selection leads to very high levels of resistance.

However, by gradually increasing the concentration of doxycycline in the medium, we were able to observe the expression of various RND efflux pumps in *B. thailandensis*. The nature of each involved pump and its level of expression appeared to be strongly related to the level of antibiotic selection pressure. The AmrAB-OprA pump was initially overproduced, allowing *B. thailandensis* to grow on media containing doxycycline at concentrations of up to 32 mg/L. As we increased the doxycycline concentration further, a jump in resistance levels to many antibiotics occurred. This shift was associated with the overexpression of another RND pump, BpeEF-OprC.

The BpeEF-OprC pump probably takes over from AmrAB-OprA at high levels of resistance to doxycycline in the resistant derivative strains, in which *amrB* expression is downregulated. Finally, the overexpression of some pumps may require a particular balance in the expression of other pumps. For example, *bpeF* expression decreased slightly with increasing *amrB* expression in E264DC16 and E264DC32, whereas expression of the *bpeB* gene, encoding a component of the third pump, decreased by 60% when *bpeF* was overexpressed. These results are consistent with the proteomics study and with the multidrug resistance phenotype of these variants: we observed a large increase in the MICs of doxycycline, chloramphenicol, fluoroquinolones and trimethoprim/sulfamethoxazole, the main antibiotics extruded by BpeEF-OprC in *B. pseudomallei* [6]. We suggest that this pump is selected in *B. thailandensis* only under conditions of extreme environmental stress due to high drug concentrations. The two overproduced RND efflux pumps at two successive stages in the doxycycline selection protocol are presumably involved in the multidrug resistance phenotype.

Expression of the BpeAB-OprB pump was reduced in doxycycline-resistant variants, whereas the AmrAB-OprA pump was overexpressed. The balance between these two pumps has been described in *B. pseudomallei* [39]. The antibiotics for which a decrease in MIC was observed, mostly ß-lactams and aminoglycosides, are the substrates of BpeAB-OprB in *B. pseudomallei* [4], [8]. The MICs of aminoglycosides (amikacin, gentamicin, tobramycin, kanamycin, netilmicin) initially increased in E264DC16 and E264DC32, and then decreased in the E264DC64 and E264DC128. This paradoxical decrease in MICs has remained unexplained in *B. pseudomallei*, as reported by Dance *et al.* in 1989 [40]. A similar phenomenon is also observed in other Gram-negative bacteria, such as *P. aeruginosa*, and hypersusceptibility to ß-lactams and aminoglycosides in multidrug-resistant strains has been attributed to a decrease in the levels of MexAB-OprM, a homolog of BpeAB-OprB [41]. Aminoglycosides are extruded principally by the AmrAB-OprA pump, but may also act as substrates of BpeAB-OprB in *B. pseudomallei*. This decrease is probably accentuated by changes in the expression of *amrB* in these variants.

We investigated the mechanisms involved, by studying two mutants lacking an efflux pump, E264Δ*amrRAB* and E264Δ*bpeEF-oprC* [21]. As for the wild-type strain, resistant variants of these mutants were obtained by selection on media with doxycycline. Thus, these two genes are dispensable for growth in the presence of doxycycline and the resistant strains compensate for their absence by expressing other genes. E264Δ*amrRAB* was susceptible to aminoglycosides and erythromycin, confirming the results obtained in *B. pseudomallei* for this pump [3], [7]. However, the MIC of doxycycline was slightly lower in this mutant. As doxycycline can induce the expression of this pump, this suggests that it is also one of its substrates. In the doxycycline-resistant derivative strains of E264Δ*amrRAB*, multidrug resistance concerned most of the antibiotics listed above, except aminoglycosides, and reflected an incremental overexpression of *bpeB* associated with a slight, but not significant downregulation of *bpeF*. BpeAB-OprB overproduction was also demonstrated in a proteomic analysis of these doxycycline-resistant derivatives (data not shown). As *bpeF* expression was slightly lower in E264Δ*amrRAB* and its doxycycline-resistant derivative strains than in the wild type (60 and 30% lower levels of expression), BpeEF-OprC production seems to be conditioned by the presence of *amrB* and the two pumps seem to be coregulated. In the absence of AmrAB-OprA, BpeAB-OprB replaces the deficient pump. However, the increase in *bpeB* expression has no impact on susceptibility to aminoglycosides in the doxycycline-resistant strains of E264Δ*amrRAB*.

In doxycycline-resistant variants derived from E264Δ*bpeEF-oprC*, we observed progressive multidrug resistance without paradoxical hypersusceptibility. In this case, both *bpeB* and *amrB* are overexpressed, and the corresponding pumps confer a high level of resistance to all antibiotics, although we cannot exclude the possibility that another efflux system is also involved. Furthermore, the selection on doxycycline of two mutants defective for the BpeEF-OprC and AmrAB pumps, respectively, shows that neither of these pumps is exclusively required for high levels of resistance to doxycycline.

The use of PAßN as an efflux pump blocker partially restored susceptibility to nalidixic acid and trimethoprim/sulfamethoxazole for all resistant variants, to doxycycline only for E264DC64 and E264DC128, but never to chloramphenicol. However, we observed antagonistic effects between PAßN and ß-lactams (cefotaxime, amoxicillin/clavulanate, imipenem and aztreonam) and aminoglycosides (gentamicin and amikacin). These antagonistic effects, which were visible in the wild-type parental strain, were also observed in all four resistant variants, with for example an 8–16 fold increase for gentamicin in the presence of PAßN. Some studies in *B. pseudomallei* have suggested that PAßN does not restore the MICs of this family of antibiotics [4]. A recent study reported an antagonistic effect of PAßN for norfloxacin, ciprofloxacin, streptomycin and ampicillin in *Y. pestis* [42].

We investigated the expression of efflux pump genes in the presence and absence of two different concentrations of PAßN. For both the *amrB* and *bpeB* genes, expression levels were significantly higher in the presence of PAßN. This induction by PAßN appeared to be dose-dependent. However, we observed no significant variation or a slight decrease in expression of the gene encoding BpeF. This pump extrudes chloramphenicol, trimethoprim/sulfamethoxazole and quinolones, such as nalidixic acid [6].

The highest levels of susceptibility restoration by PAßN were obtained with these antibiotics and BpeF overproduction, in E264DC64 and E264DC128. These observations support a dual mode of action for PAßN. PAßN can block the activity of efflux pumps and upregulate the expression of efflux pump genes. Consequently, depending on the efflux pump produced and the antibiotic used, the modulation of membrane-associated mechanism of resistance by inhibitors may differ considerably between bacterial isolates. These results may explain the increase in MICs observed for aminoglycosides and ß-lactams, but also for doxycycline, in E264DC16 and E264DC32 in the presence of PAßN.

The results presented here indicate that the overexpression of three major efflux pumps supports the emergence of multidrug resistance in *B. thailandensis* during doxycycline use and highlight the tight joint regulation of these pumps. We also found that combinations of two efflux substrates, an antibiotic and an efflux pump inhibitor, could stimulate efflux pump expression and that these substrates could have antagonistic effects rather than the expected synergistic effects. Future studies including the use of efflux pump inhibitors should take the potential inducer effect of these components on efflux pump gene expression into account.

## Supporting Information

Table S1 **Primers used in quantification studies.**
(DOC)
