# Supplementary material for: Interplay between Three RND Efflux Pumps in Doxycycline-Selected Strains of Burkholderia thailandensis
Source: PLoS One. 2013 Dec 27;8(12):e84068. doi: 10.1371/journal.pone.0084068 (PMC3873969; doi:10.1371/journal.pone.0084068)
Supplement: Table S1 — Primers used in quantification studies. (DOC) [file pone.0084068.s001.doc]

**Table S1.** Primers used in quantification studies.

| **Target** | **Oligonucleotide (5′→3′)** | **Product size (bp)** | **Annealing temperature / Annealing time** | **Cq min/max** | **Efficiency** |
| --- | --- | --- | --- | --- | --- |
| BTH_I2229 | TCTCGCCTTTCCCCACAATC | 60 | 54°C/5 s | 19/33 | 98% |
| (*rumA* 23S rRNA) | AGAGAGGGACGGCTTCTGACAC |
| BTH_I1662 | GCAAGGTTGCCGATATGATTGAC | 118 | 57°C/5 s | 18/32 | 98% |
| (*rimM* 16S rRNA) | ACGCCGACGAACGGAATC |
| BTH_I1308 | CGAAATCAACCTGCCGTACATC | 69 | 54°C/8 s | 22/34 | 105% |
| (*dnaK*) | CGGGTGATCTTCAGATTCAAGTG |
| BTH_I3073 | CACGACGACGCCGAAGAAG | 58 | 53°C/3 s | 21/34 | 97% |
| (*rpsL 30S*) | CAGACGAACCTTGGCAACCTTAC |
| BTH_I2444 | AAACGTCGAGCGGCTGATG | 74 | 55°C/5 s | 20/33 | 105% |
| (*amrB*) | TGATCTGCTTCATCGCCTTCAC |
| BTH_I0681 | TGCTCGTGCTCGCCTTCAAC | 63 | 56°C/5 s | 24/38 | 89% |
| (*bpeB*) | CGTAGTTCGCCAGGTCGTACTTG |
| BTH_II2105 | CCGAACGACAGCTACGACATGAC | 64 | 58°C/2 s | 20/34 | 93% |
| (*bpeF*) | GGCGATCCTTCACGTTGATGAG |
